# Orthodenticle homeobox OTX1 is a potential prognostic biomarker for bladder cancer

**DOI:** 10.1080/21655979.2021.1974646

**Published:** 2021-09-24

**Authors:** Lei Jiang, Zhongqiang Zuo, Jie Lin, Chuanfeng Yang

**Affiliations:** Department of Emergency, The Fourth Affiliated Hospital of Zhejiang University School of Medicine, Yiwu City, China

**Keywords:** Bladder cancer, orthodenticle homeobox 1, bioinformatics, cell behavior, prognostic biomarker

## Abstract

Bladder cancer (BC) is one of the most aggressive tumors worldwide. OTX1 (orthodenticle homeobox 1) is an important transcription factor involved in various diseases, such as cancers. The aim of this study was to further investigate the role of OTX1 in BC. In this study, differentially expressed genes (DEGs) were screened from tumor tissues and para-cancerous tissues by bioinformatics. The expression of protein and RNA was separately detected by western blotting and immunohistochemistry (IHC), and quantitative polymerase chain reaction (qPCR); cell viability and cell growth were determined by 3-(4,5-Dimethylthiazol-2-yl)-2,5-diphenyltetrazolium bromide (MTT) and clone formation assays, respectively; cell motility was measured by transwell and wound healing assays; cell cycle was measured by flow cytometry. In this study, 9 DEGs were screened out, and OTX1 was employed as a candidate gene for subsequent study. Results found that OTX1 was highly expressed in BC cells and BC tissues, which was significantly associated with poor prognosis of patients. In addition, OTX1 silencing significantly reduced cell viability, and inhibited cell growth and motility, while OTX1 overexpression got opposite results. Moreover, OTX1 co-expressed genes were enriched in cell cycle-related pathways, suggesting that the role of OTX1 in BC may be related to cell cycle, which was confirmed by flow cytometry analysis. Furthermore, *in vivo* experiments showed that OTX1 silencing significantly inhibited tumor growth in tumor-bearing mice. Taken together, our findings suggested that OTX1 may play a promotional role in BC progression.

## Introduction

Bladder cancer (BC) is one of the most aggressive tumors worldwide [1]. The 5-year overall survival (OS) for BC is reported to be maintained at about 80% [2]. Traditionally, relapse after radical cystectomy or advanced conditions is involved in the poor outcomes of patients [2]. Chemotherapy and surgery are the main treatment strategies for BC, and can effectively prolong the survival of patients with BC [3]. Unfortunately, almost of patients with advanced conditions will die from BC. Therefore, molecular biomarker contributes to the early diagnosis of disease, progression of disease, prognostic prediction [4,5], providing personalized treatment for specific patients.

Previously, tumor biomarkers were identified by genetic or mRNA profiling and screening of patients with BC [6,7]. For example, miRNA microarray demonstrated that the higher the miR-381 levels, the better the prognosis of BC patients [8]. MAFG-AS1 was identified to be a potential therapeutic target for BC through measuring the level of MAF BZIP Transcription Factor G Antisense RNA 1 (MAFG-AS1), a carcinogenic lncRNA, in paired non-tumor tissues [9].

OTX1 (orthodenticle homeobox 1), derived from OTX family proteins (OTX1, OTX2, OTX3 and CRX), plays a dominant role in the development of brain, sensory organs, early human fetal retina and breast [10–13]. Recently, OTX1 was found to be over expressed in various cancers, covering breast blastoma, breast cancer, colorectal cancer and hepatocellular carcinoma [14,15]. Further studies showed that OTX1 promoted the proliferation and migration of tumor cells [16,17]. However, the role of OTX1 in BC remains to be elucidated.

In this study, we sought to identify novel molecular markers for the prediction and diagnosis of BC and investigate the underlying molecular mechanisms. Our results showed that OTX1 is a candidate gene for the diagnosis and prediction of BC, which promoted the growth and motility of cancer cells by regulating cell cycle-related pathways, thus exhibiting carcinogenic effects.

## Material and methods

### Differentially expressed genes (DEGs)

Microarray data were downloaded from TCGA (http://cancergenome.nih.gov/) [18]. RNA-seq data of 560 samples were covered in the transcriptome profiles for BLCA BC in TCGA database (TCGA-BLCA), containing 496 tumor tissues from patients with BC and 64 normal tissues from healthy volunteers. Edger & Limma/Voom were used to analyze differentially expressed genes in TCGA transcriptome data [19], and the conditions for screening differentially expressed genes were as following: | log (FC) | >1 & FDR < 0.05 or adj. p value <0.05. The difference analysis of Chip data (GSE27448 and GSE61615) from Gene Expression Omnibus (GEO) database was carried out by Limma [20]. GeoQuery was used to process GEO data [21]. Data preprocessing included data filtering (removing all genes with 0 expression, retaining genes with a median expression>0, and averaging the expression values of repeated genes) and normalization (using normalizeBetweenArrays to normalize the log2 converted data) [22,23]. Batch effect was removed by Combat (SVA package). TCGA data was downloaded from TCGA biolinks and process [24–26]. The filtering conditions of TCGA data were as following: the genes with a expression level of 0 were removed; The genes with a expression level above the 75% of top genes were retained; The genes with a median expression >0 were retained. Tumor samples with purity >60% were screened using TCGA tumor_purity in TCGA Biolinks package [26,27]. ggplot2 was used for mapping [28], and clusterProfiler was used for KEGG-GO analysis [23,29–31]. Co-expression genes of OTX1 in TCGA bladder cancer transcriptome data were obtained through uclcan online website (Pearson correlation coefficient > 0.3). TCGA data divided into four arms by different categories (edgeR, GSE27448, GSE61615 and limma) were employed to analyze the prognosis of patients with BC, as well as the gene expression in tumor and normal tissues. Venn analysis for the four groups was performed using Venn Diagram web tool (http://bioinformatics.psb.ugent.be/webtools/Venn), and 40 genes associated with BC prognosis were picked up, of which 20 genes were up-regulated and 20 genes were down-regulated. Based on transcriptome data, the levels of DEGs in tumor tissues and normal tissues were compared.

### Patient characteristics

Tissue samples were collected from 29 patients with BC at the Fourth Affiliated Hospital of Zhejiang University School of Medicine, and verified by pathological examination. Relationship between OTX1 and clinic-pathological parameters are shown in Table 1. A total of 17 men and 12 women were included in the study. BC was diagnosed by two pathologists based on pathological evaluation. The collection of specimens was approved by the Fourth Affiliated Hospital of Zhejiang University School of Medicine. Each patient has signed the informed consent form, with available follow-up information.

**Table 1.** Relationship between OTX1 and clinic-pathological parameters

Parameters	Number of patients	OTX1 expression		*P* value
		Low (≤ median)	High (> median)	
Number	29	15	14	
Gender				
Man	17	9	8	0.876
Female	12	6	6	
Age (years)				
≥Mean (65)	17	8	9	0.550
<Mean (65)	12	7	5	
Histological grade				
Grade 1	11	8	3	0.077
Grade 2	9	5	4	
Grade 3	9	2	7	
Lymph node metastasis				
Yes	9	3	6	0.184
No	20	12	8	
TNM				
I–II	14	10	4	0.040*
III–IV	15	5	10	

### Cell culture

HCV-29 normal bladder epithelial cells derived from histologically normal urothelium [32] were propagated in Minimum Essential Medium (MEM) containing 10% FCS and antibiotics (100 U/ml penicillin, 100 fig/ml streptomycin) [33]. BC cell lines TCCSUP, SCaBER, SW780, and HT1376 were purchased from American Type Culture Collection (ATCC), and maintained in Minimum Essential Medium (MEM) with 10% fetal bovine serum (FBS). All cells were cultured in a humidified atmosphere at 37°C with 5% CO_2_.

### RT-qPCR

Total RNA (1 µg) extracted from cells were reversely transcribed into cDNA, and then real time qPCR was carried out according to the protocol of FastKing One Step RT-qPCR Kit (SYBR) (Tiangen, Beijing, China). Gene expression was normalized to that of glyceraldehyde-3-phosphate dehydrogenase (GAPDH) and quantified using the 2^−ΔΔCq^ method [34], and the primers are presented as following: OTX1 forward, 5′-CAGTGCTAATGCTTGCTC-3′ and reverse, 5′-TTGCTGTGTGCTAACGTC-3′; and GAPDH forward, 5′-GATCGTTCGAGACTCTTCC-3′ and reverse, 5′-CCTTAGCTGATGCAGCTG-3′ [35].

### Western blotting

Cells were lysed using lysis buffer containing protease inhibitor (Beyotime, Shanghai, China). After lysis, the supernatant was collected by centrifugation and used as the protein solution to be tested. Thereafter, the proteins (20 μg) were separated by 10% SDS-PAGE gels, followed by transferred onto Polyvinylidene Fluoride (PVDF) membranes. Subsequently, the membranes were blocked with defatted milk (5%), and hybridized with primary antibodies (anti-OTX1 (ab25985, 1:1000, Abcam, Cambridge, UK), anti-β-actin (ab8226, 1:1000, Abcam, Cambridge, UK), anti-CyclinE (ab33911, 1:1000, Abcam, Cambridge, UK), anti-CDK4 (ab108357, 1:2000, Abcam, Cambridge, UK), anti-CDK2 (ab32147, 1:2000, Abcam, Cambridge, UK) and anti-P21 (ab109520, 1:2000, Abcam, Cambridge, UK) at 4°C overnight. After washed with TBST three times, the membranes were then incubated with secondary antibody at room temperature for 1 h. Bands were imaged on chemiluminescence system, and analyzed using Image-Pro Plus 6.0 software (Media Cybernetics, Sarasota, USA).

### Cell transfection

HT1376 and TCCSUP cells were inoculated into a 24-well plate and then cultured until a fusion rate of 80% was reached. For OTX1 silencing, HT1376 cells were transfected with shRNA-negative control (sh-NC) and shRNAs targeting OTX1 (sh-OTX1#1 (i) 5ʹ-AAGGCATTCCGAGATCGAACTCCTGTCTC-3ʹ (sense), and 5ʹ-AAGTAACCGTTAGCTGCCAGGCCTGTCTC-3ʹ(antisense); (ii) 5ʹ-AAGTCGATCTGACTACCTGAACCTGTCTC-3ʹ (sense) and 5ʹ-AAGTTCGGCGAGCTAACTGCCCTGTCTC-3ʹ(antisense).

sh-OTX1#2 (i) 5ʹ-AAGGGCTAGTCATGCTAACTGCCTGTCTC-3ʹ (sense), and 5ʹ-AAGAATTCGATGCTAGTCTCGCCTGTCTC-3ʹ (antisense); (ii) 5ʹ-AAGTCATGGCTAGCTGATCGCCCTGTCTC-3ʹ (sense) and 5ʹ-AAGGCAGTCGTAGCTAGCGATCCTGTCTC-3ʹ (antisense) (GenePharma, Shanghai, China) using Lipofectamine 3000 (Thermo Fisher Scientific, Inc) according to the manufacturer’s instructions. For OTX1 overexpression, adenoviral vector (empty-vector) was purchased from Fenghui (Changsha, Hunan, China) and adenoviral vectors expressing OTX1 (OTX1-vector1) were constructed. After reaching a fusion rate of 80%, TCCSUP cells were transfected with empty-vector or OTX1-vector1 using PolyjetTM Transfection reagent (SignaGen, MD, USA) following instructions

### MTT assay

For cell viability, the transfected cells were plated in 96-well plates and cultured for 24 h, followed by a treatment of MTT working solution and incubation at 37°C for another 4 h. Dimethyl sulfoxide was employed to dissolve the formazan. The absorbance was determined at 490 nm with a microplate analyzer (Bio-Tek, Winooski, VT, USA).

### Clone formation assay

For cell growth, the transfected cells were maintained in complete medium. After 14 days, the cells were fixed with methanol for 30 min, and stained with 0.1% crystal violet for 20 min. After rinsed with tap water, the cells were photographed for counting.

### Transwell assay

For invasion, the transfected cells were seeded into an upper chamber (8 μm), cultured with 200 µl serum-free medium. Complete medium (600 µl) supplemented with 10% FBS was added into lower chamber (Corning, Inc.). The cells were incubated at 37°C for 24 h, followed by the fixation and staining of Crystal Violet Staining Solution (Beyotime, Shanghai, China) at RT for 30 min. Finally, cells were observed under an inverted fluorescence microscope (magnification, ×200; Olympus Corporation).

### Wound healing assay

For migration, the transfected cells (1 × 10^5^) were cultured in medium (500 µl) containing 10% FBS until a monolayer cell was formed. Monolayer cells (80–90%) were scraped using a sterile micropipette tip, and then cultured in medium without serum. Thereafter, the cells were photographed at 0 and 24 h using an inverted fluorescence microscope (Magnification, ×200; Olympus Corporation).

### Flow cytometry for cell cycle

For cell cycle, after the cells were harvested and washed with PBS, followed by staining of propidium iodide (PI, 50 mg/ml) for 30 min. Cell sorting was conducted on FACS Calibur flow cytometer (Becton Dickinson, NJ, USA).

### Animal modeling

All animal experiments were approved by Laboratory Animal Care and Use Committee of the Fourth Affiliated Hospital of Zhejiang University School of Medicine and were performed in accordance with the approved guidelines and regulations. Animal model was constructed in this study. In brief, 20 BALB/c nude mice purchased from the Shanghai Laboratory Animal Center (Shanghai, China) were housed in an environment with a constant temperature and humidity, free access to food and water. Un-transfected HT1376 cells and HT1376 cells transfected with sh-OXT1 (3 × 10^6^) were injected in the flank region of mice. After that, mice were recovered in a barrier facility under High Efficiency Particulate Arrestment (HEPA) filtration and was monitored twice per week. Tumor volume was calculated using the formula Volume = [π/6 × largest diameter × (smallest diameter) [2]] [36]. 30 days later, the mice were executed, and the tumor was removed to further investigation.

### Immunohistochemistry (IHC)

OXT1 expression in the tumors was measured by immunohistochemistry and scored according to the previous study [37,38]. Paraffin-embedded tumor tissues were cut into sections with a thickness of 5 um. Followingly, tissues were cultured with primary antibody anti-OXT1 (ab105320, 1:500, Abcam, Cambridge, UK) at 4°C overnight. After washed with tris buffered saline (TBS) solution containing 0.025% Triton X-100 twice, the samples were co-cultured with second antibody and stained with diaminobezidin (DAB) at room temperature for about 10 min. Images of tissue slices were visualized using a Nikon model Ni microscope equipped a NIS elements software and Nikon digital camera (Diagnostic Instruments, Melville NY, USA).

### Statistical analysis

Data are shown as mean ± standard deviation (SD). Clinicopathological factors were compared by χ2 test. Unpaired Student’s t-test or one-way ANOVA was employed for continuous variables. Survival analysis was carried out using Kaplan–Meier analysis and the log rank test. statistical analysis was carried out using Prism 7 (GraphPad Software, Inc.). Bioinformatics analysis was carried out using R software (Version 3.4; R Foundation for Statistical Computing). *p* < 0.05 indicates a statistically significant difference.

## Results

The purpose of this study was to investigate the role of OTX1 in BC and the potential mechanism. Bioinformatics analysis and detection of clinical samples were performed to determine the expression of OTX1 in BC patients and the effect of OTX1 expression on prognosis of BC patients was furtherly investigated. The result revealed that OTX1 was highly expressed in BC and was significantly correlated with patients’ OS and disease free survival (DFS). Therefore, we assume that OTX1 is involved in the oncogenesis of BC. To confirm this hypothesis, animal and cell experiments, combined with bioinformatics, were conducted to uncover the mechanisms of action. The results showed that OTX1 promoted cell growth and motility by regulating cell cycle in vitro, and promoted tumor growth in vivo. Our study suggests that OTX1 might be a potential prognostic biomarker for BC.

### OTX1 was overexpressed in BC

**Figure 1.**
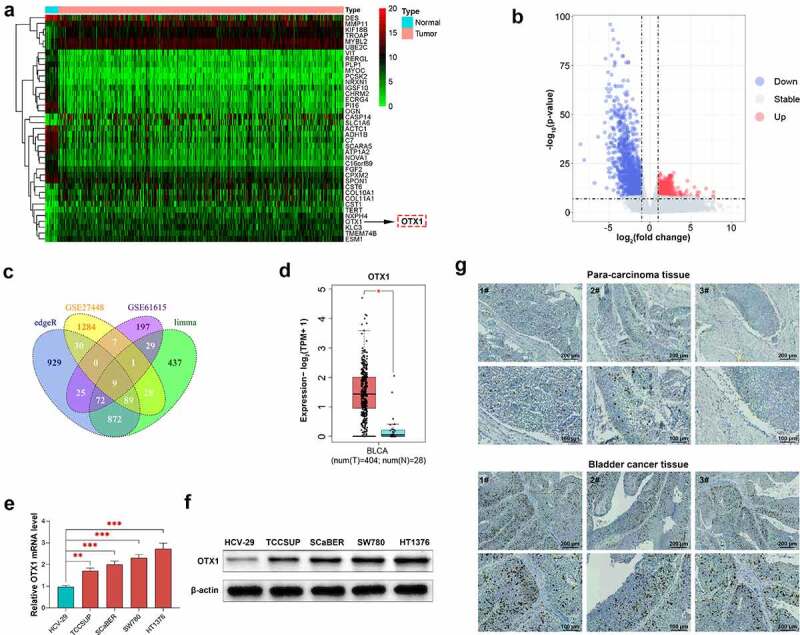
OTX1 was overexpressed in BC. (a). Hierarchical clustering analysis of different genes in Normal samples and Tumor samples. (b). Volcano plot. The red dots represent up-regulated differentially expressed genes (DEGs), while the blue dots represent down-regulated DEGs (based on > 2-fold changes). (c). Venn diagram showed the distribution of DEGs in different groups. (d). Comparison of OTX1 in normal and bladder cancer tissues based on RNA-seq. (e). The mRNA level of OTX1 was detected by qPCR in HCV-29, BT-B, EJ, HT-1376 and SCaBER. (**p < 0.05, ***p < 0.001 vs. HCV-29 cells) (f). The protein level of OTX1 was measured by western blotting in HCV-29, BT-B, EJ, HT-1376 and SCaBER. (g). The expression of OTX1 was measured by IHC in para-cancerous tissue and cancer tissue

To screen out DEGs in BC, bioinformatic analysis was performed. Nine DEGs in tumor and para-cancerous tissues were screened out (Figure 1a-c), and identified as potential predictors of poor prognosis (See Supplementary Figure S1 for details). OTX1 is a transcription factor that is commonly overexpressed in various cancers. In this study, that the expressionOTX1 was also upregulated in BC (Figure 1d). To further investigated the role of OTX1 in BC, *in vitro* experiments were carried out. As shown in Figure 1e and F, compared with that in HCV-29 cells, the expression of OTX1 in BC cells (TCCSUP, SCaBER, SW780, HT1376) was up-regulated at mRNA and protein levels. Additionally, clinical studies have shown that IHC score of tumor tissues was significantly higher than that of para-cancerous tissues (Figure 1g). Collectively, the above results indicated that OTX1 was overexpressed in BC.

### OTX1 was involved in poor prognosis of patients with BC

**Figure 2.**
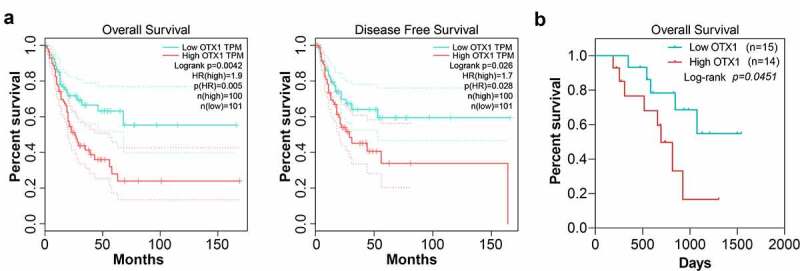
OTX1 was involved in poor prognosis of patients with BC. OS was calculated by Kaplan-Meier analysis. Patients with BC were divided into two groups based on the expression of OTX1. (a). Relationship between OTX1 expression and OS or DFS by mean optical density. (b). OS by rating level

To determine the prognostic significance of the identified genes, we examined the correlation between OTX1 and the prognosis of patients with BC. As shown in Figure 2a, OTX1 had a close relationship with OS and DFS. Besides, the expression of OTX1 in the para-carcinoma tissues and tumor tissues was detected by IHC, and the optical density value was analyzed by Image J Pro Plus software. The high and low expression of OTX1 was distinguished based on the median optical density value, and the OS was calculated by Kaplan-Meier analysis. Results showed that OTX1 had apparent correlation with OS, demonstrating that the upregulation of OTX1 was related to poor prognosis in patients with BC (Figure 2b). The detailed information about other 8 DEGs (Carboxypeptidase X, M14 family member 2 (CPXM2), Fibroblast growth factor 2 (FGF2), immunoglobulin Superfamily, Member 10 (IGSF10), neuro-oncological ventral antigen 1 (NOVA1), RERGL, scavenger receptor class A member 5 (SCARA5), F-spondin 1 (SPON1), Transmembrane protein 74B (TMEM74B)), was revealed in Supplementary Figure S2.

### *OTX1 promoted the growth and motility of BC cells* in vitro

**Figure 3.**
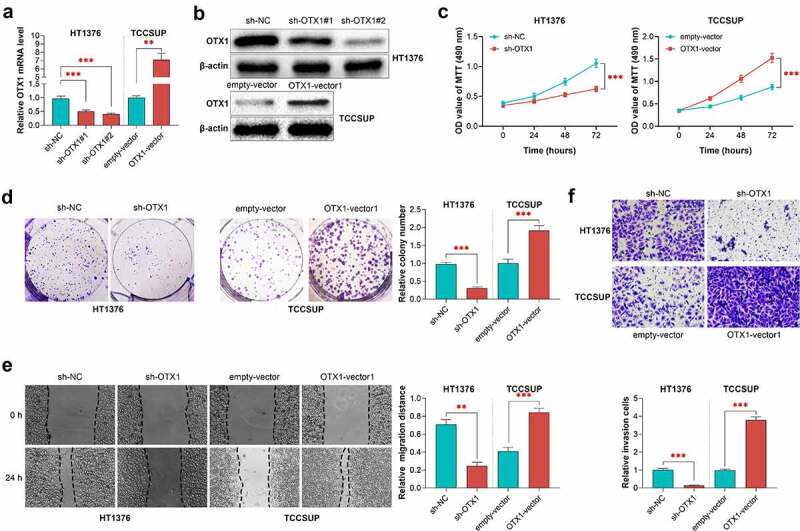
OTX1 promoted the growth and motility of BC cells *in vitro*. HT1376 cells were transfected with shRNA-negative control (sh-NC) and shRNAs targeting OTX1 (sh-OTX1#1 and sh-OTX1#2). TCCSUP cells were transfected with empty-vector or OTX1-vector1. (a). The mRNA level of OTX1 was detected by qPCR in HT1376 and TCCSUP cells. (b). The protein level of OTX1 was measured by western blotting in HT1376 and TCCSUP cells. (c). Viability of HT1376 and TCCSUP cells was measured by MTT assay. (d). Cell growth was measured by clone formation assay. (e). Cell migration was measured by Wound healing assay. (f). Cell invasion was measured by Transwell assay. (**p < 0.05, ***p < 0.001 vs. HT1376 cells transfected with sh-NC or TCCSUP cells transfected with empty-vector)

To investigate the role of OTX1 in BC *in vitro*, OTX1 was knocked down and overexpressed by shRNAs (sh-OTX1 #1 and sh-OTX1 #2) in HT1378 cells, and adenovirus (OTX1-Vector) in TCCSUP cells, respectively. As shown in Figure 3a and B, compared with sh-NC transfection, both sh-OTX1 #1 or sh-OTX1 #2 transfection decreased the expression of OTX1 in mRNA and protein level in HT1378 cells, in which, the effect of sh-OTX1 #2 transfection was more obvious. Therefore, sh-OTX1#2 was used in this study for subsequent studies. Conversely, compared with empty-vector, transfection of OTX1-vector increased the expression of OTX1 at mRNA and protein levels in TCCSUP cells, indicating that OTX1 was successfully overexpressed. Besides, the effects of OTX1 on cell growth and motility were also investigated. As shown in Figure 3c, compared with the sh-NC group, OTX1 silencing significantly reduced the viability of HT1378 cells, and inhibited the growth and the motility of HT1378 cells, but OTX1 overexpression showed opposite effects in TCCSUP cells (Figure 3e and f). Taken together, these results suggested that OTX1 promoted the growth and motility of BC cells *in vitro*.

### OTX1 promoted cell cycle progression of BC cells

**Figure 4.**
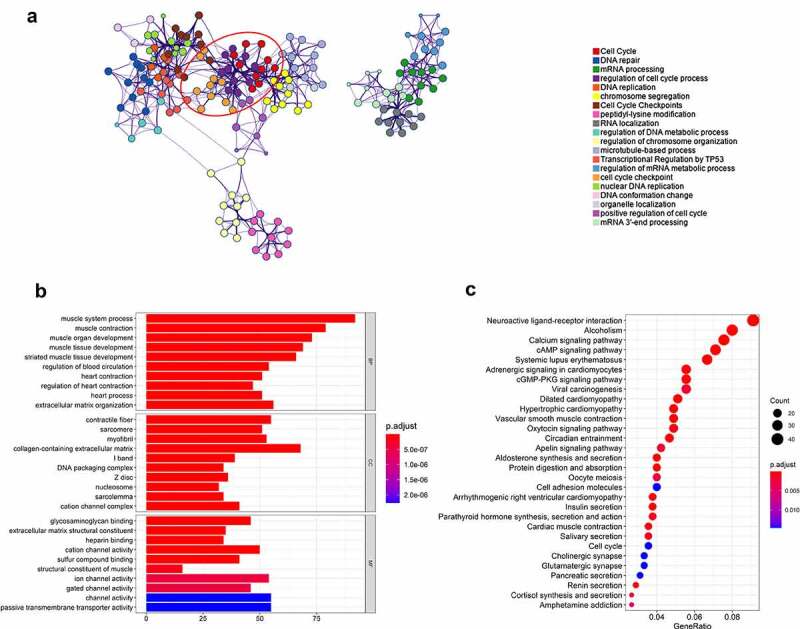
OTX1 promoted cell cycle progression of BC cells. (a). GO analysis showed that OTX1 co-expressed genes were enriched in cell cycle. (b-c). KEGG and Reactome analysis showed that cell cycle-related pathways were significantly associated with OTX1 gene in bladder cancer

**Figure 5.**
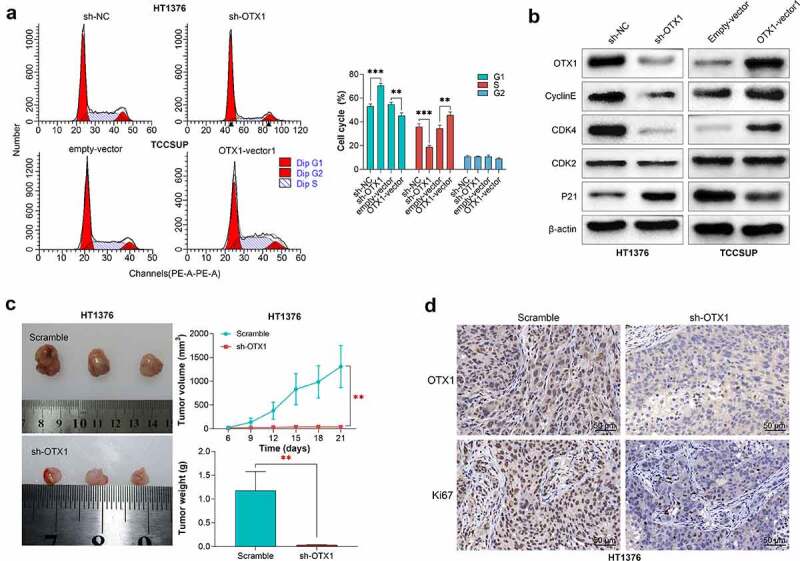
OTX1 silencing inhibited tumor growth *in vivo*. HT1376 cells were transfected with shRNA-negative control (sh-NC) and shRNAs targeting OTX1 (sh-OTX1#1). TCCSUP cells were transfected with empty-vector or OTX1-vector1. (a). Cell cycle was detected by flow cytometry analysis. (b). The protein levels of OTX1, CyclinE, CDK4, CDK2 and P21 were measured by western blotting. (c-d). Un-transfected HT1376 cells and HT1376 cells transfected with sh-OXT1 (3 × 10^6^) were injected into the flank region of mice. (c). The volume and weight of tumors. (d). The expression of OTX1 and Ki67 in tissues was detected by IHC

To further explore the potential molecular mechanism of OTX1 regulating progression of BC, OTX1 co-expression genes in TCGA bladder cancer transcriptome data were obtained through uclcan online website (Table 2). As shown in Figure 4a-C, the upregulated co-expressed genes of OTX1 were significantly related with cell cycle related pathways, suggesting that the OTX1 may play an important role in cell cycle in BC. further studies showed that OTX1 silencing increased the proportion of HT1376 cells in G0/G1 phase but decreased the proportion of cells in S phase, indicating that OTX1 silencing led to the arrest of cells in G0/G1 phase. However, overexpression of OTX1 got an opposite result, indicating that OTX1 promoted the transition from G1 phase to S phase of BC cells (Figure 5a). In addition, OTX1 silencing inhibited the expression of CyclinE and CDK4, while promoting the expression of P21, which was conversed by OTX1 overexpression (Figure 5b). Overall, the above studies suggested that OTX1 promoted cell cycle progression of BC cells.

**Table 2.** The co-expressed genes with OTX1 in BC

Gene Symbol	Gene ID	PCC
AC009501.4	ENSG00000231609.5	0.64
RP11-465I4.2	ENSG00000265643.1	0.58
ERMN	ENSG00000136541.14	0.57
C11orf16	ENSG00000176029.13	0.5
RBM38	ENSG00000132819.16	0.5
METTL13	ENSG00000010165.19	0.5
MGAT5	ENSG00000152127.8	0.5
FGF12	ENSG00000114279.13	0.5
RP11-65M17.3	ENSG00000254968.6	0.5
TMEM5	ENSG00000118600.11	0.49
AC019118.3	ENSG00000236760.1	0.49
RP11-210M15.1	ENSG00000258010.3	0.49

### *OTX1 silencing inhibited tumor growth* in vivo

To further investigate the role of OTX1 *in vivo*, animal experiments were conducted. Briefly, HT1378 cells or HT1378 cells transfected with sh-OTX1 were injected into the frank regions of the mice, respectively. Tumor volume and tumor weight was measured. As shown in Figure 5c, OTX1 silencing significantly reduced the volume and weight of tumors. Moreover, IHC assay suggested that OTX1 was successfully low-expressed, and the low expression of OTX1 decreased the expression of Ki67 (Figure 5d), thus suppressing tumor growth. In summary, OTX1 silencing inhibited tumor growth *in vivo.*

## Discussion

BC is the most common genitourinary malignancy with a high incidence in the United States [39]. Patients with low-grade BC have a low progression rate, while those with high-grade tumor have high progression rate and high cancer death rate [40]. Tumor grade is a critical factor affecting tumor progression and patient prognosis, but current studies are not enough to support the routine screening for BC [40]. In the past few years, many novel biomarkers and therapeutic targets were identified using high-throughput technology [41]. However, those biomarkers identified in BC are still insufficient to accurately diagnose disease earlier, stratify patients by risk, improve prognostic prediction, so as to provide personalized treatment for specific patients [42–44].

Previous studies have shown that type III collagen (COL3A1), Fibronectin (FN) 1, COL5A1, fibrillin 1 (FBN1), collagen type VI alpha 1 (COL6A1) and Thrombospondin-2 (THBS2) were significantly associated with a worse overall survival of BC patients [45]. By contrast, 9 DEGs (CPXM2, FGF2, IGSF10, NOVA1, RERGL, SCARA5, SPON1, TMEM74B, OTX1) were identified as potential predictors for BC in this study. Specifically, CPXM2, FGF2, IGSF10, NOVA1, RERGL, SCARA5 and SPON1 were downregulated in BC, while TMEM74B and OTX1 were upregulated. Several studies demonstrated that CPXM2, FGF2 and NOVA1 might be available biomarkers for cancers such as hepatocellular carcinoma (HCC) [46], ovarian cancer (OC) [47] and non-small cell lung cancer [48]. IGSF10, a member of the immunoglobulin superfamily, was a novel prognostic biomarker for breast cancer [49] and lung cancer [50]. Besides, comprehensive analysis showed that SCARA5 might be a suppressor gene in colorectal cancer [51]. Amazingly, in addition to the above biomarkers, RERGL, SPON1 and TMEM74B were also found to be abnormally expressed in BC. Further studies indicated that OTX1 was significantly associated with OS and DFS of BC patients. Therefore, OTX1 was selected as a candidate gene for further studies.

OTX1 is an important transcription factor involved in the development of cerebral cortex, sensory organs, early human fetal retina and mammary gland [10,52,53]. OTX1 was also reported to be involved in the progression of various tumors [54–56]. In BC, an international multi-center prospective study revealed that methylation of OTX1 togethering with FGFR3 and TERT mutations were able to anticipate the recurrence of NMIBC [57]. Besides, OTX1 methylation combined with clinical variables can be used to construct hematuria prediction model [58,59]. However, the above-mentioned studies were all based on the clinical studies. The expression of OTX1 in tumor tissues and para-cancerous tissues was evaluated by immunohistochemistry staining. In consistent with the above results, the expression of OTX1 was significantly increased in tumor tissues compared to para-cancerous tissues. In this study. OTX1 promoted the growth and motility of BC cells *in vitro*. Besides, OTX1 suppressed the growth of tumor in mice. For uncovering the mechanism of actions of OTX1, GO-KEGG analysis was performed and the results showed that OTX1 co-expressed genes were enriched in cell cycle-related pathways. Moreover, OTX1 promoted cell cycle progression, and the involvement of OTX1in cell cycle arrest was well recognized (particularly in S phase) [17,54,60], which reinforces our hypothesis that the critical role of OTX1 in BC can be attributed to the regulation of OTX1 on cell cycle. Nevertheless, further studies still need to be conducted.

## Conclusion

In conclusion, OTX1 was highly expressed in BC patients and BC cell lines, which was associated with the survival (OS and DFS) and poor prognosis of patients with BC. OTX1 silencing significantly reduced cell viability, and inhibited cell growth and motility, while OTX1 overexpression showed the opposite effect. Moreover, OTX1 silencing inhibited tumor growth i*n vivo*. GO-KEGG analysis showed that the mechanism of actions of OTX1 is related with its role in cell cycle regulation. Therefore, OTX1 might be a potential prognostic biomarker for BC.

## Supplementary Material

Supplemental Material

## Data Availability

All data generated or analyzed during this study are included in this published article.

## Highlights

1. OTX1 was overexpressed in BC
2. OTX1 was involved in poor prognosis of patients with BC
3. OTX1 promoted the growth and motility of BC cells in vitro
4. OTX1 promoted cell cycle progression of BC cells
5. OTX1 silencing inhibited tumor growth *in vivo*

## References

[1] Antoni S, Ferlay J, Soerjomataram I, et al. Bladder cancer incidence and mortality: a global overview and recent trends. Eur Urol. 2017;71(1):96–108. 27370177 10.1016/j.eururo.2016.06.010

[2] Lopez-Beltran A, Cimadamore A. Immune Checkpoint Inhibitors for The Treatment of Bladder Cancer. Cancers (Basel). 2021;13(1):131. 10.3390/cancers13010131 PMC7795541 33401585

[3] Szarvas T, Hoffmann MJ. MMP-7 Serum And Tissue Levels Are Associated with Poor Survival in Platinum-Treated Bladder Cancer Patients. Diagnostics (Basel). 2020;11(1):48. 10.3390/diagnostics11010048 PMC7824149 33396213

[4] Soria F, Krabbe LM, Todenhöfer T, et al. Molecular markers in bladder cancer. World J Urol. 2019;37(1):31–40. 10.1007/s00345-018-2503-4 PMC6510866 30259123

[5] DeGeorge KC, Holt HR, Hodges SC. Bladder cancer: diagnosis and treatment. Am Fam Physician. 2017;96(8):507–514. 29094888

[6] Yang X, Ye T, Liu H, et al. expression Profiles, Biological Functions and Clinical Significance of Circrnas in Bladder Cancer Mol Cancer. 2021;20(1):4. 10.1186/s12943-020-01300-8 PMC7780637 33397425

[7] Mati Q, Qamar S, Ashraf S, et al. Tissue nuclear matrix protein expression 22 in various grades and stages of bladder cancer. J Coll Physicians Surg Pak. 2020;30(12):1321–1325. 33397061 10.29271/jcpsp.2020.12.1321

[8] Chen D, Cheng L, Cao H. Role of microRNA-381 in Bladder Cancer Growth and Metastasis with the Involvement of BMI1 and the Rho/ROCK Axis. BMC Urol. 2021;21(1):5. 10.1186/s12894-020-00775-3 PMC7789167 33407350

[9] Tang C, Wu Y, Wang X, et al. LncRNA MAFG-AS1 regulates miR-125b-5p/SphK1 axis to promote the proliferation, migration, and invasion of bladder cancer cells. Hum Cell. 2021 Mar; 34 (2):588-597. 10.1007/s13577-020-00470-3 PMC7900043 33400245

[10] Klein WH, Li X. Function and evolution of Otx proteins. Biochem Biophys Res Commun. 1999;258(2):229–233. 10375352 10.1006/bbrc.1999.0449

[11] Omodei D, Acampora D, Russo F, et al. Expression of the brain transcription factor OTX1 occurs in a subset of normal germinal-center B cells and in aggressive Non-Hodgkin Lymphoma. Am J Pathol. 2009;175(6):2609–2617. 19893048 10.2353/ajpath.2009.090542 PMC2789631

[12] Larsen KB, Lutterodt M, Rath MF, et al. Expression of the homeobox genes PAX6, OTX2, and OTX1 in the early human fetal retina. Int J Dev Neurosci. 2009;27(5):485–492. 19414065 10.1016/j.ijdevneu.2009.04.004

[13] Pagani IS, Terrinoni A, Marenghi L, et al. The mammary gland and the homeobox gene Otx1. Breast J. 2010;16(Suppl 1):S53–6. 21050313 10.1111/j.1524-4741.2010.01006.x

[14] de Haas T, Oussoren E, Grajkowska W, et al. OTX1 and OTX2 expression correlates with the clinicopathologic classification of medulloblastomas. J Neuropathol Exp Neurol. 2006;65(2):176–186. 16462208 10.1097/01.jnen.0000199576.70923.8a

[15] Terrinoni A, Pagani IS, Zucchi I, et al. OTX1 expression in breast cancer is regulated by p53. Oncogene. 2011;30(27):3096–3103. 21478910 10.1038/onc.2011.31

[16] Yu K, Cai XY, Li Q, et al. OTX1 promotes colorectal cancer progression through epithelial-mesenchymal transition. Biochem Biophys Res Commun. 2014;444(1):1–5. 24388989 10.1016/j.bbrc.2013.12.125

[17] Li H, Miao Q. OTX1 Contributes to Hepatocellular Carcinoma Progression by Regulation of ERK/MAPK Pathway. J Korean Med Sci. 2016 Aug;31(8):1215-23. 10.3346/jkms.2016.31.8.1215 PMC4951550 27478331

[18] Li B, Cui Y, Diehn M, et al. Development and validation of an individualized immune prognostic signature in early-stage nonsquamous non-small cell lung cancer. JAMA Oncol. 2017;3(11):1529–1537. 28687838 10.1001/jamaoncol.2017.1609 PMC5710196

[19] Cui Z, Liu Y, Zhang J, et al. Super-delta2: an enhanced differential expression analysis procedure for multi-group comparisons of RNA-seq data. Bioinformatics (Oxford, England). 2021. DOI:10.1093/bioinformatics/btab155 PMC12536915 33693477

[20] Li GM, Zhang CL, Rui RP, et al. Bioinformatics analysis of common differential genes of coronary artery disease and ischemic cardiomyopathy. Eur Rev Med Pharmacol Sci. 2018;22(11):3553–3569. 29917210 10.26355/eurrev_201806_15182

[21] Davis S, Meltzer PS. GEOquery: a bridge between the Gene Expression Omnibus (GEO) and bioconductor. Bioinformatics (Oxford, England). 2007;23(14):1846–1847. 10.1093/bioinformatics/btm254 17496320

[22] Bolstad BM, Irizarry RA, Astrand M, et al. A comparison of normalization methods for high density oligonucleotide array data based on variance and bias. Bioinformatics (Oxford, England). 2003;19(2):185–193. 10.1093/bioinformatics/19.2.185 12538238

[23] Smyth GK, Speed T. Normalization of cDNA microarray data. Methods. 2003;31(4):265–273. 14597310 10.1016/s1046-2023(03)00155-5

[24] Colaprico A, Silva TC, Olsen C, et al. TCGAbiolinks: An R/Bioconductor Package for Integrative Analysis of TCGA Data. Nucleic Acids Res. 2016 May 5;44(8):e71. 10.1093/nar/gkv1507 PMC4856967 26704973

[25] Silva TC, Colaprico A, Olsen C, et al. TCGA workflow: analyze cancer genomics and epigenomics data using bioconductor packages. F1000Res. 2016;5:1542. 28232861 10.12688/f1000research.8923.2 PMC5302158

[26] Mounir M, Lucchetta M, Silva TC. New functionalities in the TCGAbiolinks package for the study and integration of cancer data from GDC and GTEx. PLoS Comput Biol. 2019;15(3):e1006701. 10.1371/journal.pcbi.1006701 PMC6420023 30835723

[27] Sha Y, Phan JH, Wang MD. Effect of low-expression gene filtering on detection of differentially expressed genes in RNA-seq data. In Annual International Conference of the IEEE Engineering in Medicine and Biology Society IEEE Engineering in Medicine and Biology Society Annual International Conference,Milan, Italy, 2015;2015:6461–6464. 10.1109/EMBC.2015.7319872 PMC4983442 26737772

[28] Cedric G. ggplot2: elegant graphics for data analysis. J R Stat Soc A Stat Soc. 2011;174:210.

[29] Bittner ML, Chen Y, Dorsel AN, et al. Microarrays: optical technologies and informatics. Microarrays. 2001;4266.

[30] Yang YH, Dudoit S, Luu P, et al. Normalization for cDNA microarray data: a robust composite method addressing single and multiple slide systematic variation. Nucleic Acids Res. 2002;30(4):e15. 11842121 10.1093/nar/30.4.e15 PMC100354

[31] Yang J, Thorne N. Normalization for two-color cDNA microarray data. Lecture Notes-Monograph Series 2003;40:403-418.

[32] Christensen B, Kieler J, Vilien M, et al. A classification of human urothelial cells propagated in vitro. Anticancer Res. 1984;4(6):319–337. 6083740

[33] Skopinska-Rózewska E, Janik P, Przybyszewska M, et al. Inhibitory effect of theobromine on induction of angiogenesis and VEGF mRNA expression in v-raf transfectants of human urothelial cells HCV-29. Int J Mol Med. 1998;2(6):649–652. 9850731 10.3892/ijmm.2.6.649

[34] Livak KJ, Schmittgen TD. Analysis of relative gene expression data using real-time quantitative PCR and the 2(-Delta Delta C(T)) method. Methods. 2001;25(4):402–408. 11846609 10.1006/meth.2001.1262

[35] Wu Q, Wang D, Zhang Z, et al. DEFB4A is a potential prognostic biomarker for colorectal cancer. Oncol Lett. 2020;20(4):114. 32863927 10.3892/ol.2020.11975 PMC7448564

[36] Tsui KH, Hsu SY, Chung LC, et al. Growth differentiation factor-15: a p53- and demethylation-upregulating gene represses cell proliferation, invasion, and tumorigenesis in bladder carcinoma cells. Sci Rep. 2015;5:12870. 26249737 10.1038/srep12870 PMC4528199

[37] Kampf C, Bergman J, Oksvold P, et al. A tool to facilitate clinical biomarker studies–a tissue dictionary based on the human protein atlas. BMC Med. 2012;10:103. 22971420 10.1186/1741-7015-10-103 PMC3523031

[38] Pontén F, Gry M, Fagerberg L, et al. A global view of protein expression in human cells, tissues, and organs. Mol Syst Biol. 2009;5:337. 20029370 10.1038/msb.2009.93 PMC2824494

[39] Farling KB. Bladder cancer: risk factors, diagnosis, and management. Nurse Pract. 2017;42(3):26–33. 10.1097/01.NPR.0000512251.61454.5c 28169964

[40] Kirkali Z, Chan T, Manoharan M, et al. Bladder cancer: epidemiology, staging and grading, and diagnosis. Urology. 2005;66(6 Suppl 1):4–34. 16399414 10.1016/j.urology.2005.07.062

[41] Xu XF, Zhou X, Wang YJ, et al. Survival-associated transcriptome analysis in ovarian cancer. Eu J Gynaecol Oncol. 2020;41(3):455.

[42] Ng K, Stenzl A, Sharma A, et al. Urinary biomarkers in bladder cancer: a review of the current landscape and future directions. Urol Oncol. 2021;39(1):41–51. 32919875 10.1016/j.urolonc.2020.08.016

[43] Wan S, Liu X, Hua W, et al. The role of telomerase reverse transcriptase (TERT) promoter mutations in prognosis in bladder cancer. Bioengineered. 2021;12(1):1495–1504. 33938397 10.1080/21655979.2021.1915725 PMC8806350

[44] Zhang F, Wang X, Hu H, et al. A hypoxia related long non-coding RNA signature could accurately predict survival outcomes in patients with bladder cancer. Bioengineered. 2021;12(1):3802–3823. 34281486 10.1080/21655979.2021.1948781 PMC8806425

[45] Shi S, Tian B. Identification of biomarkers associated with progression and prognosis in bladder cancer via co-expression analysis. Cancer Biomarkers. 2019;24(2):183–193. 30689556 10.3233/CBM-181940 PMC13082487

[46] Ye Y, An Y, Wang M, et al. Expression of carboxypeptidase X M14 family member 2 accelerates the progression of hepatocellular carcinoma via regulation of the gp130/JAK2/Stat1 pathway. Cancer Manag Res. 2020;12:2353–2364. 32280274 10.2147/CMAR.S228984 PMC7127851

[47] Meijuan W, Mao X, Wang S. Clinical significance of miR-139-5p and FGF2 in ovarian cancer. J BUON. 2021;26(3):663–669. 34268918

[48] Ludlow AT, Wong MS, Robin JD, et al. NOVA1 Regulates hTERT Splicing and Cell Growth in Non-small Cell Lung Cancer. Nat Commun. 2018;9(1):3112. 10.1038/s41467-018-05582-x PMC6079032 30082712

[49] Wang M, Dai M, Wu YS, et al. Immunoglobulin Superfamily Member 10 Is a Novel Prognostic Biomarker for Breast Cancer. PeerJ. 2020;8:e10128. 10.7717/peerj.10128 PMC7585383 33150070

[50] Ling B, Liao X, Huang Y, et al. Identification of prognostic markers of lung cancer through bioinformatics analysis and in vitro experiments. Int J Oncol. 2020;56(1):193–205. 31789390 10.3892/ijo.2019.4926 PMC6910184

[51] Liu J, Zeng ML, Shi PC, et al. SCARA5 is a novel biomarker in colorectal cancer by comprehensive analysis. Clin Lab. 2020;66:7. 10.7754/Clin.Lab.2019.191015 32658413

[52] Acampora D, Barone P, Simeone A. Otx genes in corticogenesis and brain development. Cereb Cortex. 1999;9(6):533–542. 10498271 10.1093/cercor/9.6.533

[53] Wada S, Sudou N, Saiga H. Roles of Hroth, the ascidian otx gene, in the differentiation of the brain (sensory vesicle) and anterior trunk epidermis in the larval development of Halocynthia roretzi. Mech Dev. 2004;121(5):463–474. 15147764 10.1016/j.mod.2004.03.017

[54] Chen FY, Zhou ZY, Zhang KJ, et al. Long Non-coding RNA MIR100HG Promotes the Migration, Invasion and Proliferation of Triple-negative Breast Cancer Cells by Targeting the miR-5590-3p/OTX1 Axis. Cancer Cell Int. 2020;20:508. 10.1186/s12935-020-01580-6 PMC7568413 33088216

[55] Yang CY, Wang L, Mu DC, et al. OTX1 is a novel regulator of proliferation, migration, invasion and apoptosis in lung adenocarcinoma. Eur Rev Med Pharmacol Sci. 2020;24(18):9497–9510. 33015792 10.26355/eurrev_202009_23035

[56] Tu XP, Li H, Chen LS, et al. OTX1 Exerts an Oncogenic Role and Is Negatively Regulated by miR129-5p in Laryngeal Squamous Cell Carcinoma. BMC Cancer. 2020;20(1):794. 10.1186/s12885-020-07279-1 PMC7446126 32838760

[57] Beukers W, van der Keur KA, Kandimalla R, et al. FGFR3, TERT and OTX1 as a urinary biomarker combination for surveillance of patients with bladder cancer in a large prospective multicenter study. J Urol. 2017;197(6):1410–1418. 28049011 10.1016/j.juro.2016.12.096

[58] Beukers W, Kandimalla R, van Houwelingen D, et al. The use of molecular analyses in voided urine for the assessment of patients with hematuria. PLoS One. 2013;8(10):e77657. 24143252 10.1371/journal.pone.0077657 PMC3797079

[59] van Kessel KE, Beukers W, Lurkin I, et al. Validation of a DNA Methylation-Mutation Urine Assay to Select Patients with Hematuria for Cystoscopy. J Urology. 2017;197(3 Pt 1):590–595. 10.1016/j.juro.2016.09.118 27746284

[60] Qin SC, Zhao Z, Sheng JX, et al. Dowregulation of OTX1 attenuates gastric cancer cell proliferation, migration and invasion. Oncol Rep. 2018;40(4):1907–1916. 30066897 10.3892/or.2018.6596 PMC6111461

