# Supplementary material for: Orthodenticle homeobox OTX1 is a potential prognostic biomarker for bladder cancer
Source: Bioengineered. 2021 Sep 24;12(1):6559–71. doi: 10.1080/21655979.2021.1974646 (PMC8806575; doi:10.1080/21655979.2021.1974646)
Supplement: Supplemental Material [file KBIE_A_1974646_SM8996.zip › supplementary/Supplementary Figure legends.docx]

**Supplementary Figure 1** Abnormal expression of the other 8 DEGs (CPXM2, FGF2, IGSF10, NOVA1, RERGL, SCARA5, SPON1, TMEM74B)

**Supplementary Figure 2** A. Relationship between the expression of DEGs and OS by mean optical density. B. Relationship between the expression of DEGs and DFS by mean optical density.
