# Supplementary figures and images for: Orthodenticle homeobox OTX1 is a potential prognostic biomarker for bladder cancer
Source: Bioengineered. 2021 Sep 24;12(1):6559–71. doi: 10.1080/21655979.2021.1974646 (PMC8806575; doi:10.1080/21655979.2021.1974646)

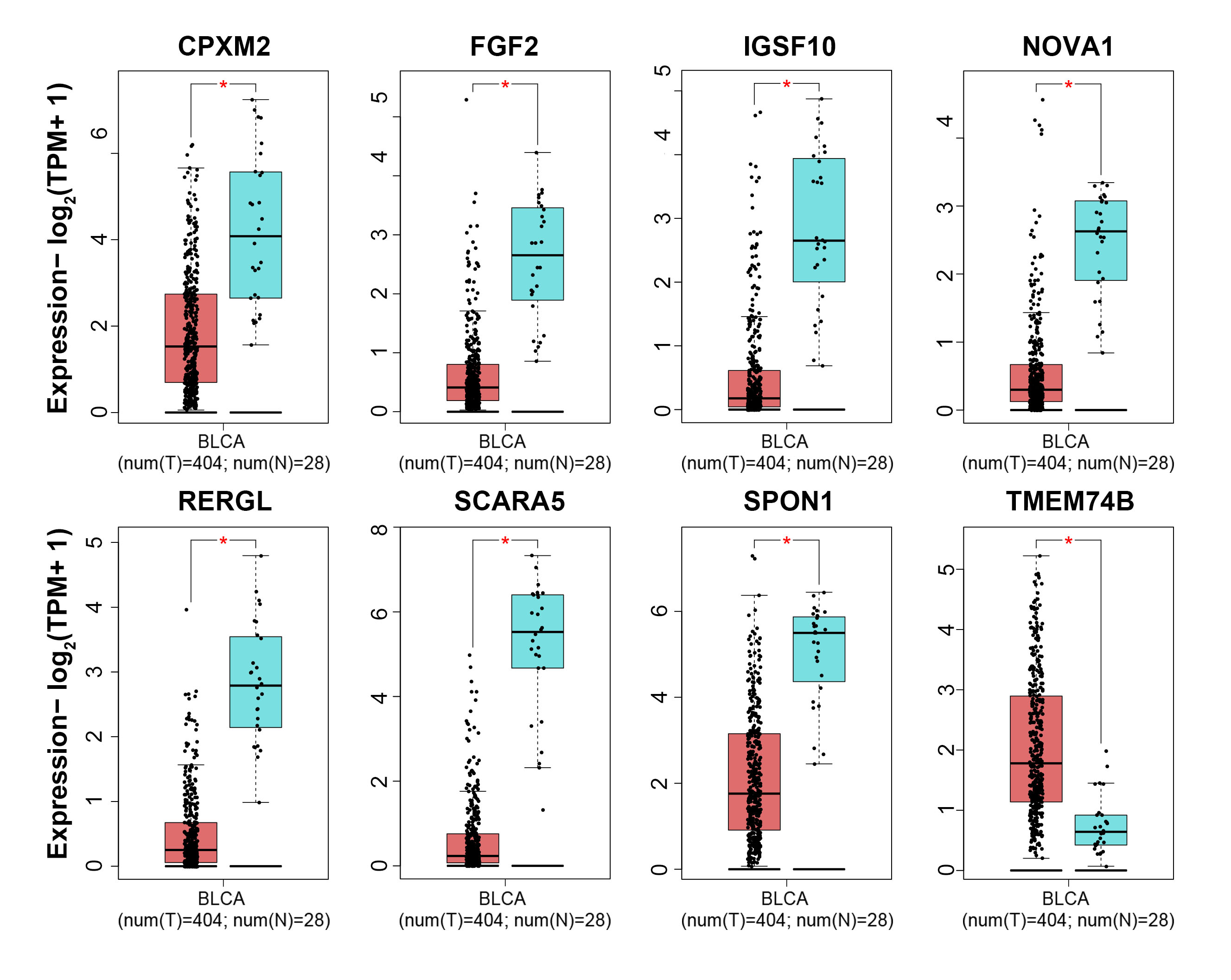

Supplement: Supplemental Material [file KBIE_A_1974646_SM8996.zip › supplementary/figs1 (1).jpg]

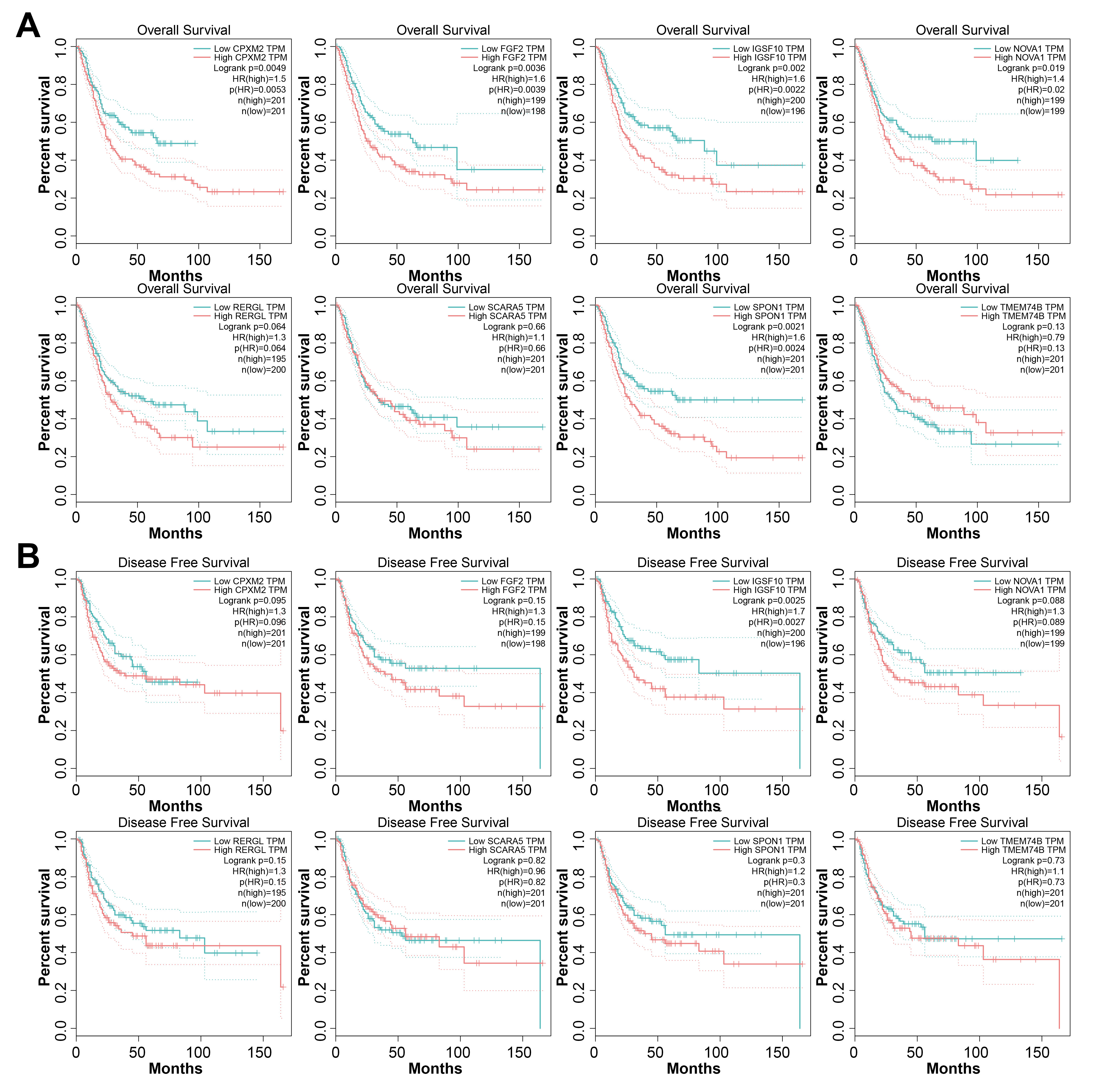

Supplement: Supplemental Material [file KBIE_A_1974646_SM8996.zip › supplementary/figs2 (1).jpg]
